# Elevated Adrenocorticotropic Hormone, Hypercortisolism, and Marked Hypernatremia

**DOI:** 10.7759/cureus.19714

**Published:** 2021-11-18

**Authors:** Elias Wassel, Chukwuemeka A Umeh, Curren Giberson, Simran K Anand, Anphong Nguyen, Hayden Porter, Prithi Choday, Harpreet Kaur, Ankur Kundu, Jose Penaherrera

**Affiliations:** 1 Internal Medicine, St. George's University School of Medicine, West Indies, GRD; 2 Internal Medicine, Hemet Global Medical Center, Hemet, USA; 3 Radiology, Hemet Global Medical Center, Hemet, USA

**Keywords:** hyperglycemia, hypokalemia, desmopressin, case report, hypernatremia, cushing syndrome, hypercortisolism, acth

## Abstract

We present a case of refractory hypernatremia in a patient with elevated adrenocorticotropic hormone (ACTH) and hypercortisolism. Cortisol’s effect in tissues results in various outcomes, from maintaining blood pressure to increasing serum glucose levels. In addition, cortisol, cortisone, and aldosterone activate mineralocorticoid receptors with the same affinity; therefore, the activation of mineralocorticoid receptors by elevated cortisol levels leads to increased sodium reabsorption, increased potassium secretion, and metabolic alkalosis. Hypernatremia in our patient was initially refractory to fluid replacement but was eventually corrected with intravenous fluid and desmopressin. Overall, we suggest that hypercortisolism should be considered a differential diagnosis in hypernatremia refractory to fluids replacement.

## Introduction

Cushing syndrome results from prolonged exposure to glucocorticoids and presents with specific signs and symptoms. Adrenocorticotropic hormone (ACTH) dependent Cushing syndrome is divided into Cushing disease from pituitary secretion and ectopic ACTH syndrome from an extra-pituitary secretion. Ectopic ACTH production accounts for 10%-20% of the cases of ACTH-dependent Cushing syndrome [1]. Common sites of paraneoplastic ACTH secretion include small cell lung carcinoma, islet cell tumor, bronchial carcinoid, and pheochromocytoma. At its physiological level, cortisol has a weak mineralocorticoid effect. However, at increased cortisol concentrations, the enhanced mineralocorticoid effect of cortisol leads to additional transcription of mineralocorticoid receptors (MRs) in the distal convoluted tubules of renal tubules, causing increased sodium reabsorption and potassium secretion [2,3]. We present a case in which hypercortisolism due to elevated ACTH caused refractory hypernatremia.

## Case presentation

A 74-year-old male with a history of two transient ischemic attacks was brought into our facility for altered mental status after being found unconscious by a relative. His social history was significant for 55 pack-years of smoking. Due to his mentation on arrival, no further history was obtained. Vital signs were normal on admission. Physical examination on admission showed non-responsiveness to sternal rub, dry mucous membranes, decreased skin turgor, and thick white plaques around the corners of his mouth. Examination of the extremities showed 1+ bilateral lower leg edema, and pulmonary examination revealed right-sided wheezes and rales.

On admission, the patient had an elevated blood glucose of 662 mg/dL (normal: 70-110 mg/dL), an anion gap of 15, ß-hydroxybutyrate of 1.86 mmol/L (normal: 0.02-0.27 mmol/L), point-of-care (POC) lactate of 3.3 mmol/L (normal: 0.5-2.0 mmol/L), serum potassium of 3.8 mEq/L, urine volume of 850 mL, and no ketones on urinalysis. The arterial blood gas (ABG) showed a pH of 7.48, HCO_3_ of 24.6, and PCO_2_ of 33 meq/L. The slightly elevated anion gap on admission is likely multifactorial. It could have been caused by the increase in the negative charge for albumin and enhanced production of lactate seen in metabolic alkalosis [4]. Conversely, it is also possible that scant ketones, which are produced in a hyperosmolar hyperglycemic state, as seen by mildly elevated β-hydroxybutyrate and the absence of ketones in the urine, could have contributed to the elevated anion gap [5].

**Figure 1:**
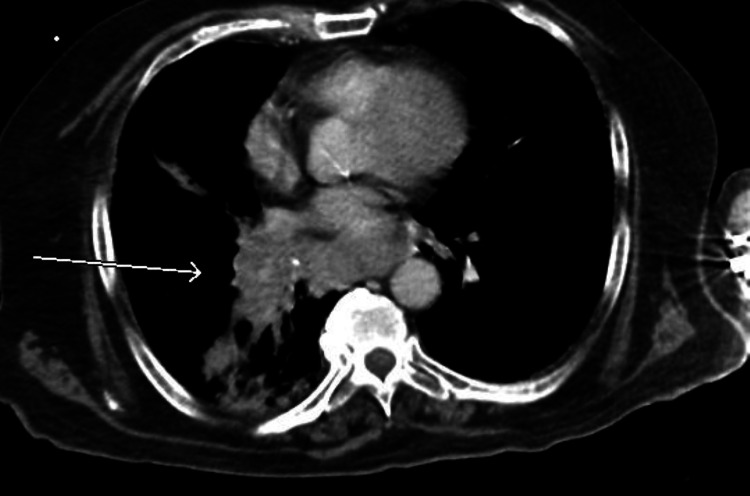
CT of the chest (axial view) showing a mediastinal mass

**Figure 2:**
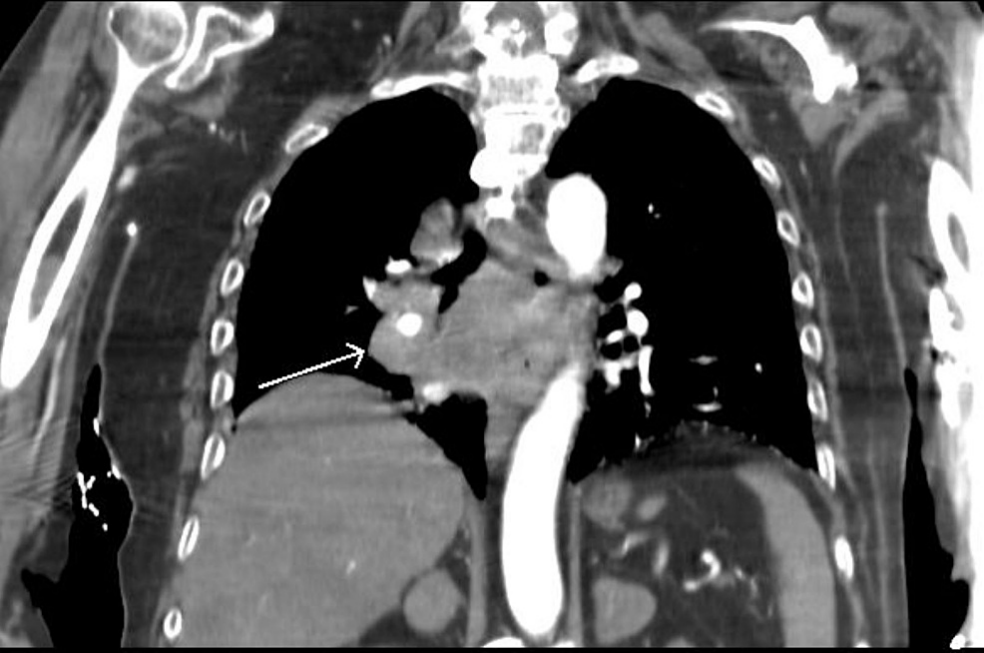
CT of the chest (coronal view) showing a mediastinal mass

**Figure 3:**
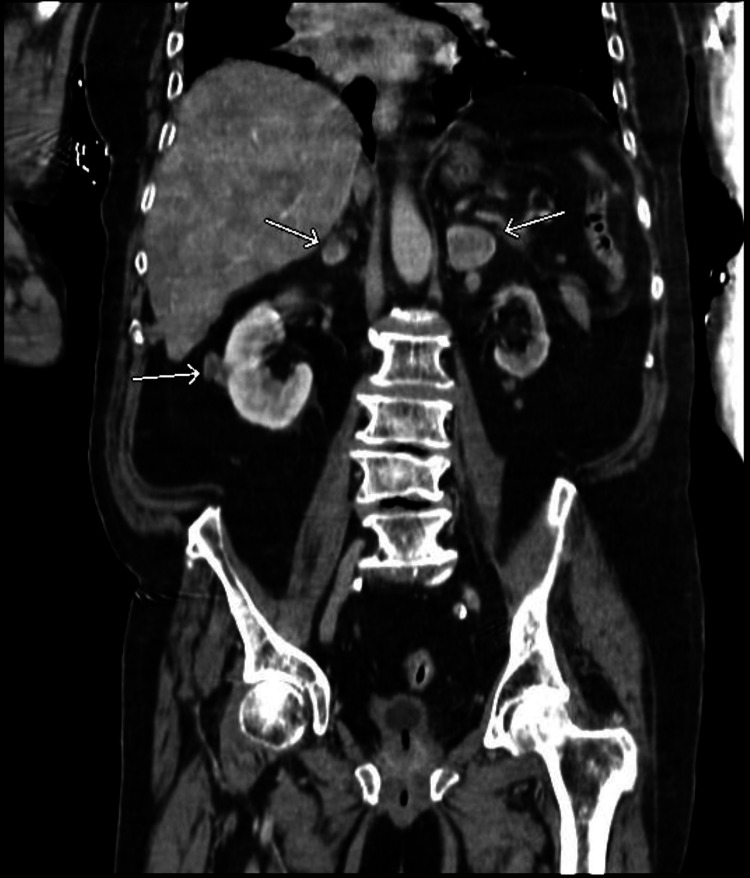
CT of the abdomen (coronal view) showing adrenal nodules and kidney nodular densities

**Figure 4:**
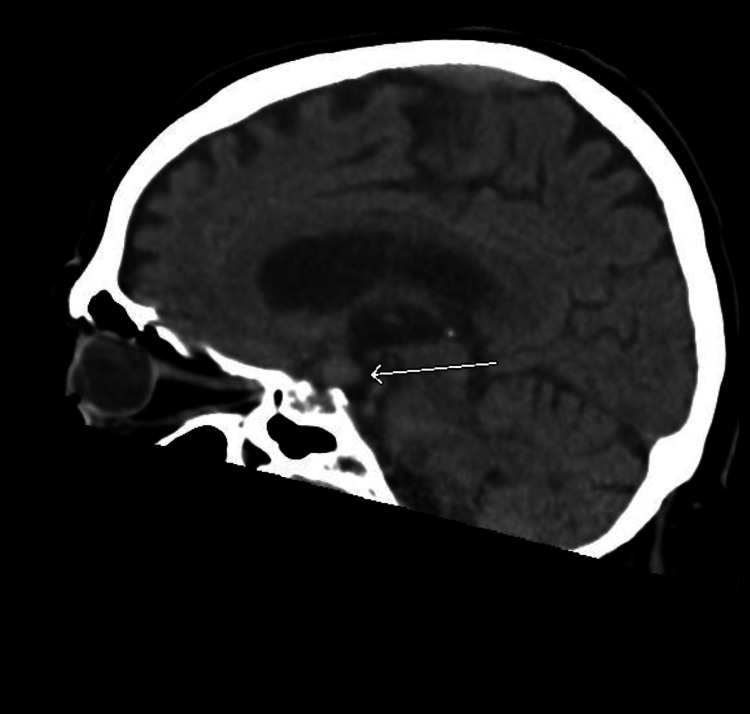
CT of the brain (sagittal view) showing a suprasellar mass

**Figure 5:**
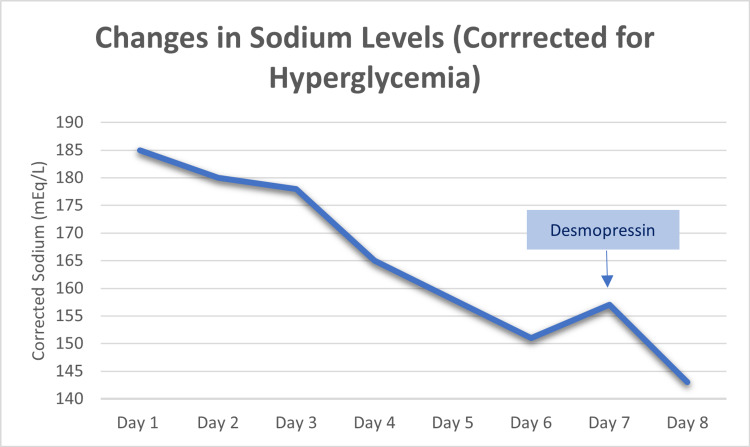
Graph showing the trend of the sodium levels

Additionally, the patient had hypernatremia (sodium of 185 mEq/L corrected for hyperglycemia), acute kidney injury (blood urea nitrogen [BUN] of 49 mg/dL, creatinine [Cr] of 1.55 mg/dL, from a previous baseline Cr of 1.01 mg/dL), and hemoconcentration (hemoglobin [Hb] 17.1 g/dL, hematocrit [Hct] 51.6%). Chest X-ray indicated a potential right lower lung zone infiltrate. Computed tomography (CT) of the chest indicated right lower lobe mass or consolidation, with multiple mediastinal and hilar masses as well as enlarged left axillary nodes compatible with lymphadenopathy, suggestive of malignancy and metastatic disease (Figures 1, 2). CT of the abdomen showed heterogeneous liver attenuation but could not differentiate between nonocclusive disease and metastatic disease. Adrenal nodules and kidney nodular densities were also present (Figure 3). CT of the brain showed a suprasellar mass measuring 1.2 x 1.1 x 0.9 cm (Figure 4). The patient was started on half normal saline and insulin, and the acute kidney injury significantly improved on day 3; however, no significant improvement in sodium level was noticed after correction for elevated glucose levels (Figure 5 and Table 1).

**Table 1:** Glucose and sodium level changes

	Day 1	Day 2	Day 3	Day 4	Day 5	Day 6	Day 7	Day 8
Sodium (mEq/L)	172	177	178	162	154	149	153	138
Corrected sodium (mEq/L)	185	180	178	165	158	151	157	143
Glucose (mg/dL)	662	209	95	222	266	174	284	308

**Figure 6:**
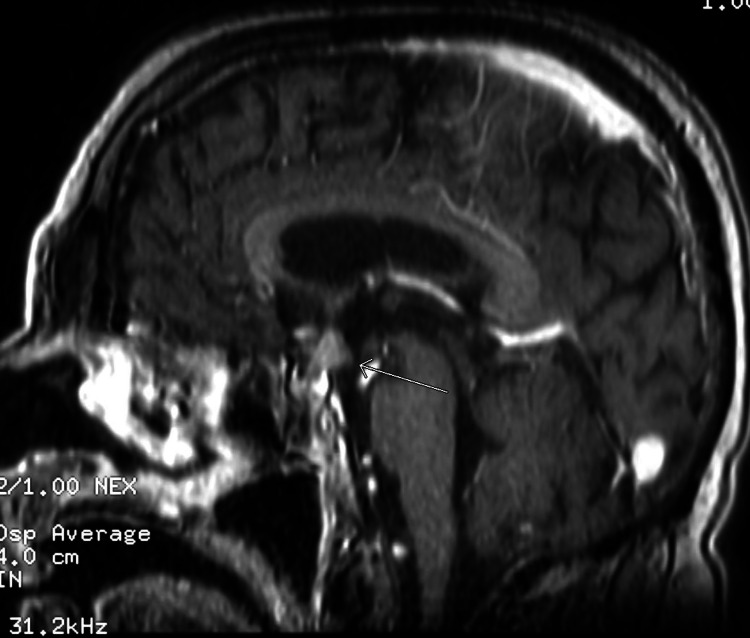
MRI of the brain (sagittal view) showing a contrast-enhancing lesion of the optic chiasm

Additional investigations for the refractory hypernatremia showed a urine osmolarity of 699 (normal: 50-1,400), serum osmolarity of 360 (normal: 280-301), urine sodium of 10 mEq/L (normal: 20 mEq/L), serum AM cortisol of 61.3 ug/dL (normal AM: 6.2-19.4 ug/dL), 1-mg dexamethasone suppression test cortisol of 6.06 ug/dL (normal: <1.8ug/dL) and ACTH of 228 pg/mL (normal: 7.2-63.3 pg/mL) (Table 2). MRI of the brain revealed a 1.1 x 1.2 x 1.1 cm enhancing lesion of the optic chiasm without evidence of mass effect (Figure 6) and small lymph nodes in deep portions of the bilateral parotid glands that were potential metastasis. Throughout the admission, the patient’s arterial blood gas (ABG) showed a primary respiratory alkalosis with secondary metabolic alkalosis (pH of 7.48, HCO_3_ of 24.6, PCO_2_ of 33 meq/L), which was persistent until discharge (pH of 7.48, HCO_3_ of 27.6 meq/L, PCO_2_ of 37 mmHg). Additional endocrine investigations demonstrated an follicle-stimulating hormone (FSH) of 0.3 miU/L (normal: 1.5-12.4 miU/L), luteinizing hormone (LH) < 0.3 miU/L (normal: 1.7-8.6 miU/L), testosterone of 27 ng/dL (normal: 264-916 ng/dL), thyroid-stimulating hormone (TSH) of 0.01 u/iU/L (normal: 0.34-5.60 u/iU/L), and prolactin of 9.7 ng/mL (normal: 4.0-15.2 ng/mL). The plan was to obtain a lung biopsy to determine whether the mass was cancerous or not and to conduct a high dexamethasone suppression test for ectopic ACTH production. However, the patient declined further workup or treatment, opting for hospice instead.

**Table 2:** Laboratory values on admission CO_2_, carbon dioxide; BUN, blood urea nitrogen; WBC, white blood cells; ACTH, adrenocorticotropic hormone; FSH, follicle-stimulating hormone; LH, luteinizing hormone; TSH, thyroid-stimulating hormone

	Patient	Normal
Sodium (mEq/L)	172	135-145
Potassium (mEq/L)	3.8	3.5-5.1
Chloride (meq/L)	123	98-107
CO_2_ (mEq/L)	26	21-31
BUN (mg/dL)	49	7-25
Creatinine (mg/dL)	1.55	0.7-1.3
WBC (10^3^/mL)	9.1	3.6-11.2
Hemoglobin (g/dL)	17.1	12.5-16.3
Hematocrit (%)	51.6	36.7-47.1
Blood glucose (mg/dL)	662	70-110
ß-hydroxybutyrate (mmol/L)	1.86	0.02-0.27
Urine osmolarity	699	50-1400
Serum osmolarity	360	280-301
Urine sodium (mEq/L)	10	20
Serum AM cortisol (ug/dL)	61.3	6.2-19.4
ACTH (pg/mL)	228	7.2-63.3
FSH (miU/L)	0.3	1.5-12.4
LH (miU/L)	<0.3	1.7-8.6
Testosterone (ng/dL)	27	264-916
TSH (u/iU/L)	0.01	0.34-5.60
Prolactin (ng/mL)	9.7	4.0-15.2

Despite initial fluid resuscitation with half normal saline followed by dextrose water, the hypernatremia did not correct. The hypernatremia later resolved after the patient was started on desmopressin on day 7 of admission. The patient was sent home on hospice and was advised to increase his water intake.

## Discussion

Cortisol induces gluconeogenesis and glycogenolysis and decreases insulin secretion, leading to increased glucose levels, as seen in our patient. Furthermore, elevated cortisol levels activate MRs in the renal tubules, which enhance the transcription of epithelial sodium channels (eNAC) and Na+/K+ ATPase channels [6]. The increased sodium and potassium channels in the luminal membrane and increased activity of the Na+/K+ ATPase channel in the basolateral membrane result in increased sodium reabsorption and potassium excretion [6,7]. The sustained activation of MRs leads to increased reabsorption of HCO_3_, leading to metabolic alkalosis [8]. These changes result in hypernatremia, hypokalemia, and metabolic alkalosis, as was seen in our patient. Further workup for Cushing disease was not conducted due to the patient’s request for hospice. However, the absence of Cushing stigmata, such as truncal/central obesity, round face, supraclavicular fat, and a buffalo hump, made Cushing disease unlikely. Consequentially, this increased our suspicion for ectopic ACTH production as a paraneoplastic syndrome. Treatment for ectopic ACTH production includes tumor excision when the source is known [9]. However, when the source of ectopic ACTH production is unknown, pharmacological treatment to suppress cortisol suppression is used, such as metyrapone or ketoconazole [9].

Our patient presented with hyperglycemia, hypernatremia, and metabolic alkalosis secondary to hypercortisolism. He received intravenous fluids without improvement in his hypernatremia. Therefore, after seven days, desmopressin was started to correct the hypernatremia. Desmopressin (1-deamino-8-D-arginine vasopressin; DDAVP) therapy decreased sodium levels by stimulating antidiuretic hormone (ADH) receptors in renal collecting ducts, leading to increased water absorption. In addition, DDAVP leads to increased water absorption through V2 receptors in the renal collecting duct, which leads to a decrease in serum sodium level [10]. Alternative treatments include eNAC channel blockers such as amiloride [11]; however, due to the need to regularly monitor potassium levels, this was not a choice for our patient who was discharged home on hospice.

## Conclusions

MRs have the same affinity for aldosterone, cortisol, and corticosterone. Thus, in hypercortisolism, MRs enhance sodium reabsorption in exchange for hydrogen and potassium ions in the renal tubules, resulting in hypernatremia, hypokalemia, and metabolic alkalosis. Therefore, we suggest that hypercortisolism should always be considered in cases of refractory hypernatremia, especially if a patient is hyperglycemic or hypokalemic. In summary, we presented a case of excess cortisol-induced hypernatremia, which was refractory to initial fluid therapy.
